# Pan-immune-inflammation value predicts proliferative diabetic retinopathy in patients with diabetes mellitus and atherosclerotic cardiovascular disease

**DOI:** 10.1590/1806-9282.20250489

**Published:** 2025-09-19

**Authors:** Alparslan Kurtul, Bengi Ece Kurtul, Ahmet Elbeyli

**Affiliations:** 1 Hatay Mustafa Kemal University, Tayfur Ata Sökmen Faculty of Medicine, Department of Cardiology – Antakya, Turkey.; 2 Hatay Mustafa Kemal University, Tayfur Ata Sökmen Faculty of Medicine, Department of Ophthalmology – Antakya, Turkey.

**Keywords:** Diabetes mellitus, Retinopathy, Atherosclerosis, Cardiovascular, Immune, Inflammation

## Abstract

**OBJECTIVE:**

Diabetic retinopathy exhibits a higher risk of atherosclerotic cardiovascular disease-related complications. Proliferative diabetic retinopathy is the most serious complication of diabetic retinopathy and can lead to total visual loss. Given that inflammation plays a key role in diabetic retinopathy, using blood-derived indexes could be a feasible way to predict the proliferative diabetic retinopathy. The aim of this study was to assess the effectiveness of pan-immune-inflammation value in predicting proliferative diabetic retinopathy in type 2 diabetes mellitus patients and atherosclerotic cardiovascular disease.

**METHODS:**

This retrospective study included 234 patients with type 2 diabetes mellitus and atherosclerotic cardiovascular disease. The study population was classified into three groups according to severity of diabetic retinopathy: no-diabetic retinopathy, non-proliferative diabetic retinopathy, and proliferative diabetic retinopathy groups. Complete blood counts were obtained, and neutrophil/lymphocyte ratio, platelet/lymphocyte ratio, and pan-immune-inflammation value ([neutrophils×platelets×monocytes]÷lymphocytes) were calculated. A receiver operating characteristic curve was performed to analyze the ability of inflammatory markers for proliferative diabetic retinopathy.

**RESULTS:**

The mean age was 56.6±7.8 years, and there were 109 men and 125 women. The median (interquartile range) pan-immune-inflammation value levels in the no-diabetic retinopathy, non-proliferative diabetic retinopathy, and proliferative diabetic retinopathy groups were 213 (137–325), 240 (157–297), and 375 (318–540), respectively (p<0.001). Multivariate analysis revealed that the pan-immune-inflammation value (OR 1.066; 95%CI 1.051–1.072; p=0.001), total cholesterol (OR 1.055; p=0.018), and hemoglobin (OR 0.145; p=0.036) were independent risk factors of proliferative diabetic retinopathy in patients with type 2 diabetes mellitus and atherosclerotic cardiovascular disease.

**CONCLUSION:**

Our study revealed a significant association between increased pan-immune-inflammation value levels and high proliferative diabetic retinopathy risk in type 2 diabetes mellitus patients and atherosclerotic cardiovascular disease.

## INTRODUCTION

Diabetes mellitus (DM) complications include retinopathy, perhaps neuropathy, and nephropathy, and enhance the morbidity and mortality risk in patients with atherosclerotic cardiovascular disease (ASCVD)^1^. Diabetic retinopathy (DR) is a common microvascular complication of DM and remains the leading cause of preventable visual loss in developed countries^2^. The damage of DR starts with non-proliferative DR (NPDR) and progresses to an advanced stage as proliferative DR (PDR)^3^. PDR is the final and most critical stage of DR^4^. PDR may cause progressive loss of peripheral and central vision. Therefore, it's critical to identify patients who could have PDR. Inflammation is a key mechanism in the development of DR^5^. Research in recent years has emphasized the role of inflammatory molecules with angiogenic and apoptotic activity in the pathogenesis of PDR^6–8^. The association between neutrophil-to-lymphocyte ratio (NLR), platelet-to-lymphocyte ratio (PLR), and the presence and severity of DR in patients with T2DM has been reported^9^.

The pan-immune-inflammation value (PIV), which has been recently introduced as a blood count–based immunoinflammatory marker, has prognostic significance in different cancers and cardiovascular diseases^10–15^. From an ophthalmological point of view, until now, no study has demonstrated the clinical importance of PIV in terms of DR in patients with type 2 DM (T2DM) and asymptomatic ASCVD.

Therefore, we aimed to investigate whether there is an association of PIV with the presence and severity of DR in T2DM, considering that inflammation involves the initiation and progression of DR in T2DM. We also aimed to compare the predictive value of the PIV and other systemic inflammatory markers based on complete blood cell count, including NLR and PLR for PDR.

## METHODS

The present study was a retrospective cohort study conducted at the Hatay Mustafa Kemal University Hospital, from 2020 to 2022, and patients with T2DM and asymptomatic ASCVD who applied to the ophthalmology outpatient clinic for an assessment of DR were included. The study originally included 245 patients. The exclusion criteria were as follows: (1) patients without the assessment of neutrophil, platelet, monocyte, and lymphocyte count; (2) patients with ocular diseases such as uveitis and glaucoma; (3) patients with a history of ocular surgery such as vitrectomy and intravitreal injections; and (4) systemic diseases (except for hypertension and DM), such as systemic infectious/inflammatory disorders and cancer. Ultimately, the study comprised 234 eligible patients. A patient was diagnosed as having ASCVD if there were ischemic findings on a resting 12-lead electrocardiogram (e.g., abnormal Q waves, ST segment depression, or inverted T waves) or a history of angina, myocardial infarction, or coronary revascularization (percutaneous coronary intervention/coronary artery bypass grafting surgery), peripheral artery disease, ischemic stroke, or transient ischemic attack, according to international guidelines.

Complete blood counts were obtained, and NLR, PLR, and PIV levels were calculated from the results of the automated hematology analyzers. The PIVs were computed following Fucà and colleagues’ original formula, utilizing the counts of neutrophils, platelets, monocytes, and lymphocytes obtained at the time of outpatient clinic admission: PIV=[neutrophils×platelets×onocytes]÷lymphocytes^10^. For every patient, clinical characteristics (age, gender, hypertension, body mass index), and biochemical data, including serum creatinine, C-reactive protein (CRP), fasting serum glucose, as well as hemoglobin A1c were also collected from the electronic medical records.

Data from all patients’ detailed ophthalmic exams including visual acuity, biomicroscopic anterior and posterior segment examination, and intraocular pressure measurements were evaluated. The presence and stages of the DR in the patients with DM were graded into three stages based on the clinical examination: no-DR (NoDR), NPDR, and PDR. Fundus photography, fundus fluorescein angiography, and optical coherence tomography were also used to confirm the status of DR. The Early Treatment Diabetic Retinopathy Study criteria were utilized to describe various stages of DR^16^.

The present study was conducted in accordance with the principles of the Declaration of Helsinki. It was also approved by the Local Ethics Committee of Hatay Mustafa Kemal University Hospital (2024/09). The written informed consent was waived owing to the retrospective nature of the study.

### Statistical analysis

SPSS software (version 21.0; IBM, Armonk, NY, USA) was used for statistical analysis. Continuous variables were presented as mean and standard deviation (SD) or median (25–75 interquartile range [IQR]) according to distribution. A one-way analysis of variance (ANOVA) test was used for the comparison of normally distributed data, and the Kruskal-Wallis test was used for the comparison of non-normally distributed data. Categorical variables were presented as numbers and proportions, and the chi-square test was used for comparisons among groups. ROC curve analysis was used to evaluate the predictive ability of systemic inflammatory indicators to distinguish PDR from NPDR and NoDR. Their predictive value was evaluated by comparing the area under the ROC curve (AUC) for each variable. A p-value of <0.05 was significant, and a value <0.10 was considered a trend. To distinguish independent risk factors for PDR, all variables with a p-value not greater than 0.10 in univariate analysis were entered into multivariate analysis using logistic regression. However, the neutrophil, platelet, monocyte, lymphocyte, NLR, and PLR were interpreted as a consequence of similar variables, and so they were not included in the regression analysis.

## RESULTS

**Figure 1:**
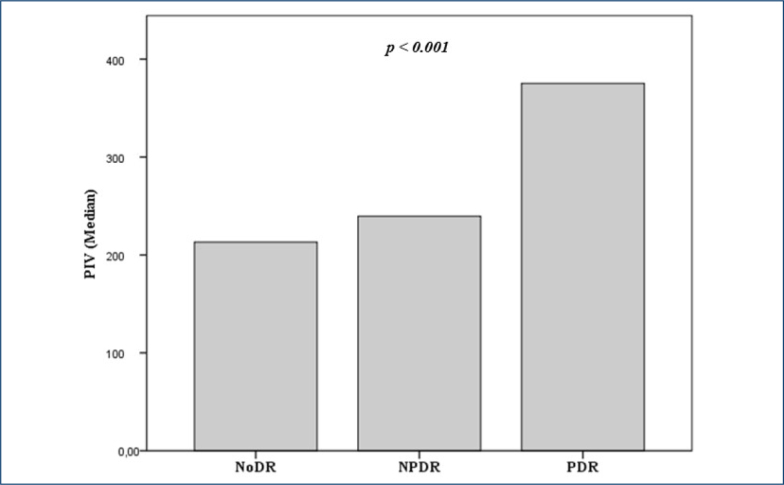
Comparison of median pan-immune-inflammation value levels among no-diabetic retinopathy, non-proliferative diabetic retinopathy, and proliferative diabetic retinopathy groups.

A total of 234 patients were included in this study: 156 in the NoDR group, 60 in the NPDR group, and 18 in the PDR group. The mean age was 56.6±7.8 years, and there were 109 men and 125 women. The age, sex, body mass index, and hypertension differences among the groups were insignificant. The clinical characteristics, biochemical data, and complete blood count data of the three groups are shown in Table 1. The differences in the laboratory parameters of the groups were statistically significant, except for PIV, diabetes duration, total cholesterol, hemoglobin, neutrophil, hemoglobin A1c, and serum creatinine. The median (25–75 IQR) PIV levels in the NoDR group, NPDR group, and PDR group were 213 (137–325), 240 (157–297), and 375 (318–540), respectively (p<0.001; Figure 1).

**Table 1:** Comparison of clinical and laboratory data among the groups.

Variables	Diabetic retinopathy			p-value
	No (n=156)	Nonproliferative (n=60)	Proliferative (n=18)	
Age (years)	56±8	58±7	59±9	0.304
Gender (Male/women)	79/77	23/37	07-Nov	0.212
Body mass index (kg/m^2^)	32.3±7.9	30.4±5.2	30.0±5.1	0.148
Hypertension	73 (46.8%)	34 (56.7%)	10 (55.6%)	0.381
Diabetes duration (years)	6 (2–10)	11 (5–15)	16 (7–21)	<0.001
Total cholesterol (mg/dL)	179±41	184±54	243±50	<0.001
LDL cholesterol (mg/dL)	114±37	117±36	138±45	0.120
HDL cholesterol (mg/dL)	39±8	40±9	36±11	0.334
Triglyceride (mg/dL)	165 (129–235)	159 (107–267)	180 (134–304)	0.739
Hemoglobin (g/dL)	13.7±1.7	13.3±1.7	11.2±1.0	<0.001
Lymphocyte count (x10^9^/L)	2.47±0.77	2.34±0.73	2.05±0.67	0.067
Neutrophil count (x10^9^/L)	4.64±1.39	4.80±1.91	6.16±1.19	<0.001
Monocyte count (x10^9^/L)	0.45±0.13	0.48±0.18	0.50±0.15	0.216
Platelet count (x10^9^/L)	256±64	260±62	275±59	0.470
PIV	213 (137–325)	240 (157–297)	375 (318–40)	<0.001
Fasting glucose (mg/dL)	147 (114–184)	151 (103–205)	162 (87–245)	0.990
Hemoglobin A1c (%)	8.25±2.04	8.87±2.06	9.96±2.72	0.005
Serum creatinine (mg/dL)	0.76 (0.65–0.90)	0.80 (0.75–1.05)	1.03 (0.84–2.03)	<0.001
C-reactive protein (mg/L)	3.36 (3.11–7.58)	4.80 (3.11–7.71)	8.09 (3.16–12.5)	0.184

LDL: low-density lipoprotein; HDL: high-density lipoprotein; PIV: pan-immune-inflammation value. Note: Values are presented as number (%), mean±SD, or median (25–75 interquartile range).

**Figure 2:**
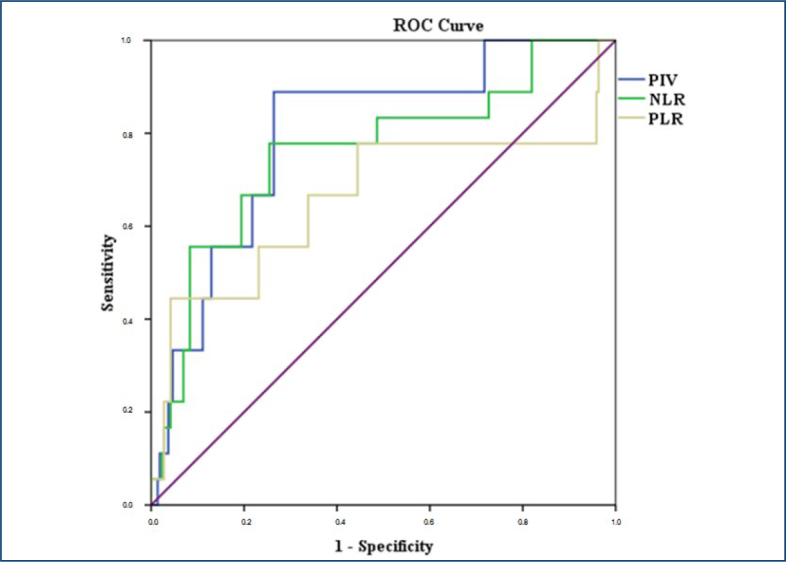
Receiver operating characteristic curves of neutrophil/lymphocyte ratio, platelet/lymphocyte ratio, and pan-immune-inflammation value to distinguish proliferative diabetic retinopathy from nonproliferative diabetic retinopathy and no-diabetic retinopathy.

According to ROC curve analysis, PIV, NLR, and PLR were significant predictors of PDR (p<0.001, p<0.001, p=0.024, respectively). Notably, the AUC of the PIV for predicting PDR was higher than those of the NLR and PLR (0.800 vs. 0.760 and 0.660, respectively), and had the highest diagnostic value (Table 2 and Figure 2).

**Table 2:** Sensitivity, specificity, and likelihood ratios of inflammatory variables for predicting a proliferative diabetic retinopathy.

Parameter	Area under ROC curve	Cutpoint	Sensitivity	Specificity	95%CI	p-value
PIV	0.800	>311	89%	75%	0.700–0.900	<0.001
NLR	0.760	>2.36	78%	74%	0.632–0.889	<0.001
PLR	0.660	>123	67%	66%	0.491–0.829	0.024

PIV: pan-immune-inflammation value; NLR: neutrophil/lymphocyte ratio; PLR: platelet/lmphocyte ratio; ROC: receiver operating characteristic; CI: confidence interval.

In the multivariate forward logistic regression analysis after adjusting for all potential risk factors, we found that PIV (OR 1.066; 95%CI 1.051–1.072; p=0.001), together with total cholesterol (OR 1.055; p=0.018) and hemoglobin (OR 0.145; p=0.036), was still independently associated with PDR in patients with T2DM with concomitant asymptomatic ASCVD. Univariate and multivariate regression analyses are presented in Table 3.

**Table 3:** Univariate and multivariate regression analyses for the presence of proliferative diabetic retinopathy in type 2 diabetes mellitus patients and asymptomatic atherosclerotic cardiovascular disease.

Variable	Univariate analysis		Multivariate analysis	
	Odds ratio (95%CI)	p-value	Odds ratio (95%CI)	p-value
Body mass index	0.959 (0.876–1.050)	0.371		
Diabetes duration	1.170 (1.080–1.268)	<0.001	1.219 (0.919–1.618)	0.168
Hemoglobin A1c	1.334 (1.073–1.658)	0.010	1.896 (0.967–3.717)	0.063
PIV	1.002 (1.001–1.003)	0.003	1.066 (1.051–1.072)	0.001*
Serum creatinine	2.562 (1.386–4.736)	0.003	4.038 (0.805–20.269)	0.090
Total cholesterol	1.027 (1.011–1.043)	0.001	1.055 (1.009–1.103)	0.018*
Hemoglobin	0.368 (0.242–0.560)	<0.001	0.145 (0.024–0.878)	0.036*
C-reactive protein	1.069 (0.984–1.162)	0.113		

PIV: pan-immune-inflammation value; CI: confidence interval.

* Represents the independent factors by multivariate analysis.

## DISCUSSION

In the present study, we evaluated if there is a relationship between PIV, as a novel inflammatory marker, and PDR in patients with T2DM and ASCVD. We found that PIV was independently and positively associated with PDR. Besides, increasing serum total cholesterol and declining hemoglobin levels were also independently associated with the occurrence of PDR in total patients.

The stage of PDR is characterized by the presence of abnormal new blood vessels, the so-called new vessels, at the optic disc or elsewhere on the retina^17^. PDR can progress to high-risk characteristics, and in severe cases, fibrovascular membranes grow over the retinal surface, and tractional retinal detachment with sight loss can occur, despite treatment. Although most, if not all, individuals with diabetes will develop DR if they live long enough, fortunately, only some progress to the sight-threatening PDR stage^18^. Additionally, the retinal microvasculature reflects pathology in the systemic small vessels including the coronary microcirculation. In general, risk factors for vascular disease are common to both the coronary and retinal arteries. Previous studies have confirmed that diabetic patients with DR exhibit a higher risk of all-cause mortality and cardiovascular disease–related death compared to those without DR^19^. Moreover, in previous studies, Xie et al.^20^ and Yamada et al.^21^ reported that patients with T2DM and PDR have an increased risk of ASCVD. Therefore, it is imperative to identify certain interventions aimed at mitigating the incidence of PDR and practical noninvasive biomarkers for predicting the risk of PDR.

Several blood-derived markers are used as systemic inflammatory status indicators in various ocular diseases. Recently, Dascalu et al.^22^ investigated the association between NLR, PLR, and the presence and severity of DR in patients with T2DM. They reported that NLR and diabetic nephropathy were associated with higher rates of PDR. On the other hand, Zeng et al.^23^ found that PLR was an independent risk factor for DR, but there was no correlation between the severity of DR and the increase in NLR or PLR.

The clinical importance of PIV in the ophthalmology area has not been investigated to date. Thus, understanding the specific relationship of PIV with PDR in the presence of ASCVD in T2DM patients is clinically relevant. According to our study results, we showed that the higher PIV is significantly and independently associated with the odds of developing PDR among patients with T2DM and ASCVD.

The biological mechanism underlying the predictive value of PIV in PDR remains unclear. Normally, the retina is an immune-privileged region that is divided by blood–retinal barriers that restrict the exchange of proteins and fluids. However, long-term exposure to chronic low-grade inflammation and oxidative stress can cause the destruction of the blood–retina barrier, triggering bleeding, exudation, and edema of the retinal tissue, which mark the onset of DR^24,25^. The PIV level may be more stable than independent neutrophil, platelet, monocyte, and lymphocyte cell levels because of the balance among the cells, which is less affected by various physiological and pathological processes.

There are some limitations in our study. First, the retrospective observational design of our study cannot determine the causality. The effect of PIV on PDR and cardiovascular outcomes still needs to be confirmed in future randomized clinical trials. Nonetheless, the result may help shed light on the importance of the PIV in this population at risk. Second, blood count tests were conducted only at baseline, and there was a lack of long-term assessment. The dynamic change of PIV may further modify their association with PDR and cardiovascular outcomes. Third, the population of the study was only limited to Turkey, thus warranting further investigation into its generalizability across other countries.

## CONCLUSION

In conclusion, for the first time, we showed that increased PIV levels were significantly associated with a higher risk of PDR in patients with T2DM with ASCVD. This association persisted after adjusting for various confounding factors. Our findings highlight the importance of PIV in the inflammation assessment of patients with PDR.

## Data Availability

The datasets generated and/or analyzed during the current study are available from the corresponding author upon reasonable request.

## References

[1] Barkoudah E, Skali H, Uno H, Solomon SD, Pfeffer MA (2012). Mortality rates in trials of subjects with type 2 diabetes. J Am Heart Assoc.

[2] Saeedi P, Petersohn I, Salpea P, Malanda B, Karuranga S, Unwin N (2019). Global and regional diabetes prevalence estimates for 2019 and projections for 2030 and 2045: results from the International Diabetes Federation Diabetes Atlas, 9th edition. Diabetes Res Clin Pract.

[3] Nawaz IM, Rezzola S, Cancarini A, Russo A, Costagliola C, Semeraro F (2019). Human vitreous in proliferative diabetic retinopathy: characterization and translational implications. Prog Retin Eye Res.

[4] Chaudhary S, Zaveri J, Becker N (2021). Proliferative diabetic retinopathy (PDR). Dis Mon.

[5] Tang J, Kern TS (2011). Inflammation in diabetic retinopathy. Prog Retin Eye Res.

[6] Shi Q, Wang Q, Wang Z, Lu J, Wang R (2023). Systemic inflammatory regulators and proliferative diabetic retinopathy: a bidirectional Mendelian randomization study. Front Immunol.

[7] Batsos G, Christodoulou E, Christou EE, Galanis P, Katsanos A, Limberis L (2022). Vitreous inflammatory and angiogenic factors on patients with proliferative diabetic retinopathy or diabetic macular edema: the role of Lipocalin2. BMC Ophthalmol.

[8] Andrés-Blasco I, Gallego-Martínez A, Machado X, Cruz-Espinosa J, Lauro S, Casaroli-Marano R (2023). Oxidative stress, inflammatory, angiogenic, and apoptotic molecules in proliferative diabetic retinopathy and diabetic macular edema patients. Int J Mol Sci.

[9] Dascalu AM, Georgescu A, Costea AC, Tribus L, El Youssoufi A, Serban D (2023). Association between neutrophil-to-lymphocyte ratio (NLR) and platelet-to-lymphocyte ratio (PLR) with diabetic retinopathy in type 2 diabetic patients. Cureus.

[10] Fucà G, Guarini V, Antoniotti C, Morano F, Moretto R, Corallo S (2020). The pan-immune-inflammation value is a new prognostic biomarker in metastatic colorectal cancer: results from a pooled-analysis of the valentino and TRIBE first-line trials. Br J Cancer.

[11] Kucuk A, Topkan E, Ozkan EE, Ozturk D, Pehlivan B, Selek U (2023). A high pan-immune-inflammation value before chemoradiotherapy indicates poor outcomes in patients with small-cell lung cancer. Int J Immunopathol Pharmacol.

[12] Feng J, Wang L, Yang X, Chen Q, Cheng X (2024). Pretreatment pan-immune-inflammation value (PIV) in predicting therapeutic response and clinical outcomes of neoadjuvant immunochemotherapy for esophageal squamous cell carcinoma. Ann Surg Oncol.

[13] Wu B, Zhang C, Lin S, Zhang Y, Ding S, Song W (2023). The relationship between the pan-immune-inflammation value and long-term prognoses in patients with hypertension: National Health and Nutrition Examination study, 1999-2018. Front Cardiovasc Med.

[14] Inan D, Erdogan A, Pay L, Genc D, Demırtola AI, Yıldız U (2023). The prognostic impact of inflammation in patients with decompensated acute heart failure, as assessed using the pan-immune inflammation value (PIV). Scand J Clin Lab Invest.

[15] Şen F, Kurtul A, Bekler Ö (2024). Pan-immune-inflammation value is independently correlated to impaired coronary flow after primary percutaneous coronary intervention in patients with ST-segment elevation myocardial infarction. Am J Cardiol.

[16] Early Treatment Diabetic Retinopathy Study Research Group (1991). Ophthalmology.

[17] Xu H, Chen M (2017). Diabetic retinopathy and dysregulated innate immunity. Vision Res.

[18] Perais J, Agarwal R, Evans JR, Loveman E, Colquitt JL, Owens D (2023). Prognostic factors for the development and progression of proliferative diabetic retinopathy in people with diabetic retinopathy. Cochrane Database Syst Rev.

[19] Cheung N, Wong TY (2008). Diabetic retinopathy and systemic vascular complications. Prog Retin Eye Res.

[20] Xie J, Ikram MK, Cotch MF, Klein B, Varma R, Shaw JE (2017). Association of diabetic macular edema and proliferative diabetic retinopathy with cardiovascular disease: a systematic review and meta-analysis. JAMA Ophthalmol.

[21] Yamada T, Itoi T, Kiuchi Y, Nemoto M, Yamashita S (2012). Proliferative diabetic retinopathy is a predictor of coronary artery disease in Japanese patients with type 2 diabetes. Diabetes Res Clin Pract.

[22] Dascalu AM, Georgescu A, Costea AC, Tribus L, El Youssoufi A, Serban D (2023). Association between neutrophil-to-lymphocyte ratio (NLR) and platelet-to-lymphocyte ratio (PLR) with diabetic retinopathy in type 2 diabetic patients. Cureus.

[23] Zeng J, Chen M, Feng Q, Wan H, Wang J, Yang F (2022). The platelet-to-lymphocyte ratio predicts diabetic retinopathy in type 2 diabetes mellitus. Diabetes Metab Syndr Obes.

[24] Hudson N, Campbell M (2019). Inner blood-retinal barrier regulation in retinopathies. Adv Exp Med Biol.

[25] Sigurdardottir S, Zapadka TE, Lindstrom SI, Liu H, Taylor BE, Lee CA (2019). Diabetes-mediated IL-17A enhances retinal inflammation, oxidative stress, and vascular permeability. Cell Immunol.

